# Correlation between knowledge on transmission and prevention of HIV/STI and proficiency in condom use among male migrants from Africa and Middle East evaluated by a Condom Use Skills score using a wooden penile model

**DOI:** 10.1186/s13104-017-2520-1

**Published:** 2017-06-19

**Authors:** Fabio Zoboli, Domenico Martinelli, Mariantonietta Di Stefano, Massimo Fasano, Rosa Prato, Teresa Antonia Santantonio, Jose’ Ramòn Fiore

**Affiliations:** 1 Clinic of Infectious Diseases, Department of Clinical and Experimental Medicine, University of Foggia, Viale Pinto 5, 71100 Foggia, Italy; 2 Sector of Hygiene, Department of Medical and Surgical Sciences, University of Foggia, Foggia, Italy

**Keywords:** Condom, Migrants, HIV, STI, Skills score, Penile model

## Abstract

**Background:**

Migrants in Italy are prevalently young adults, with a higher risk of sexual transmitted infections (STI) and HIV infection. Promoting consistent as well as correct use of condoms could reduce failure rate due to their improper use. The aim of our study was to evaluate Condom Use Skills among a migrant population recently landed in Italy, hosted in a government center for asylum seekers.

**Methods:**

The study sample was composed of 80 male migrants. Sanitary trained interviewers submitted a questionnaire to participants to investigate age, provenience, marital status, educational level and knowledge about transmission and prevention of HIV/STI. Then, we assessed participants’ level of condom use skill with the Condom Use Skills (CUS) measure by using a wooden penile model. The interviewer filled in a checklist and assigned 1 point for correct demonstration of each behavior that may prevent condom failure during sex.

**Results:**

Participants’ median age was 26 years and the sample was composed of 54 migrants from sub-Saharan Africa and 26 from Middle East. Most of them were married, with a lower middle level of education, up to 8 or 5 years. Half of the sample achieved the highest score in the questionnaire and our CUS showed a large number of people with middle high score classes. The Spearman’s rho was 0.30, therefore answers to the questionnaire and CUS score appeared correlated (p < 0.05). In the multivariate model, to have a higher CUS score resulted to be associated to be older than 26 years (p < 0.05), with a higher level of education (p = 0.001), and a higher score in the questionnaire (p < 0.05). There were no significant differences in the level of CUS between single or married men and between African and Middle Asian migrants of the sample.

**Conclusions:**

Our study shows that educational level influences the quality of knowledge and awareness about STI/AIDS and contribute to correct condom use. Since the half of participants had a low educational level and linguistic problems, the risk of missing campaigns messages or misunderstanding informative materials increases. Direct observation of condom-application on penile model may offer realistic assessment of application skills in these individuals.

**Electronic supplementary material:**

The online version of this article (doi:10.1186/s13104-017-2520-1) contains supplementary material, which is available to authorized users.

## Background

Nowadays there are 5 million regular migrants in Italy (8% of whole Italian population) and a conspicuous number of foreigners without residence permit [1]. The size of migratory flows increases year by year (43,000 in 2013, 170,000 in 2014) [2].

Migrants are prevalently young sexually active adults, who seldom know the most frequent STIs or preventive measures. The lack of distribution of informative materials in foreigner languages, the exiguous number of sanitary operators fluently speaking different languages or cultural mediators helping people to approach health problems, are the reasons of this poor knowledge [3].

As a consequence, in 2014 annual HIV incidence was higher in migrant population in Italy than autochthonous population (19.2/100,000 inhabitants vs 4.7/100,000 inhabitants), regardless of age. As well, an increased proportion of known HIV cases in Italy are amongst migrants (from 11% in 1992 to 27.1% in 2014 of) [4].

In Italy, HIV-infected migrants prevalently are men (57.6%) coming from Africa (45.1%) and the median age of first HIV diagnosis is analogous between men and women (39 vs 36 years old), both in reproductive age. For this reason, heterosexual contacts are the most frequent way to acquire HIV among migrant population, while homosexuals (20%) and injective drug users (2.3%) are less common among foreigner patients [4]. A primary goal of educational interventions is to influence people sexually active people to lower their risk for HIV and other STIs through the regular use of condoms [5–7], but literature on condom use among migrant population is scarce.

Furthermore, HIV prevention interventions have focused on self reported increase of condom use, while only a few studies have included a demonstration of the proper method of using a condom or an evaluation of Condom Use Skills [8].

However, although a lot of studies had tried to inform the development of multilevel strategies for addressing HIV risk among migrants, a recent review had shown that the study locations were Africa (23%), the Americas (26%), Europe (7%), South East Asia (21%), and Western Pacific (24%), but none of these papers involved population from Middle East [23]. In the last few years this geographic area has become a critical region because of wars and ethnic persecution and the number of asylum seekers increases every year, but this migrant population was less studied.

For these reasons, the aim of our study is to evaluate Condom Use Skills among migrant population that is located in our local community. Indeed, 250,000 regular foreigners live in the province of Foggia, Apulia [1], and another consistent number of migrants is hosted in a government center of asylum seekers (Borgo Mezzanone, Manfredonia, FG). We want to study the general level of Condom Use Skills among Borgo Mezzanone’s guests, using a wooden penile model and a lubrificated condom, because the consciousness of the real knowledge of preventive measures could suggest tailored campaigns for these increasing populations to reduce sexual transmitted infections, such as HIV.

## Patients, materials and methods

The study sample consisted of migrant asylum seekers recently landed in Italy that were temporarily located in Borgo Mezzanone, government center of asylum seekers (Foggia, Italy). Participants were enrolled in the study if they were able to understand and provide written informed consent. We included only males, because of cultural and religious reasons, for the first time in a European Country and we ruled out migrants under 18 years old.

Interviewers trained by health personnel submitted an anonymous questionnaire to participants: the first part investigated age, provenience and marital status of the guests. We also inquired the education level, that was subdivided in: Elementary (E) if participants have studied up to 5 years; Middle (M) up to 8 years; Superior (S) more than 8 years; University (U) if migrants have attended university courses.

To analyze sexual risk behavior knowledge, the second part of the interview schedule was designed to obtain information in a variety of areas related to HIV/AIDS and other STIs, their transmission and prevention (Additional file 1). Data were gathered regarding the participant’s history of sexual activity, possible disorders or diseases, condom attitudes and behaviors, and perceived Condom Use Skills. Participants gain one point for each correct answer, from 0 (all wrong answers) to 20 (no mistakes). We thus divided participants in 4 marks, based on distribution of scores: (A) 16–20 points, (B) 10–15 points, (C) 6–10 points, (D) 0–5 points.

After the questionnaire, we assessed participants’ level of condom use skill with the Condom Use Skills measure, that comprised 10 steps. Each Condom Use Skills item is a single, discrete and directly observable behavior that may prevent condom failure during sex or facilitate proper condom disposal after the act.

Participants were given lubrificated condoms and a wooden penile model and asked to demonstrate the steps for using and then discarding a condom, without any prompt by the interviewer. Therefore, the latter used a checklist and assigned one point for correct demonstration of each of the following steps by participants:

1. Check expiration date on package of condom.
2. Open package carefully (not roughly or with their teeth).
3. Check condom for damage.
4. Determine direction in which condom rolls.
5. Roll condom correctly downward on the penile model.
6. Roll condom to base of penis.
7. Remove air from condom.
8. Leave space at tip of condom.
9. Turn to the side and withdraw condom.
10. Take care to avoid spilling and dispose of in trash.

Each item was coded either yes (1 point) or no (0 point). Therefore, the score ranged from 0 to 10. The Condom Use Skills score was composed of 4 classes too, determined by distribution of actual scores: (A) 9–10 points, (B) 6–8 points, (C) 3–5 points, (D) 0–2 points.

## Data analysis

Characteristics of the study sample were reported in term of absolute and relative frequencies. For the age, we considered the median to divide the sample in two groups (subjects <median age, subjects ≥median age).

To assess the level of concordance between the score to the questionnaire and the skill score, we calculated the Spearman’s rho, considering p < 0.05.

To evaluate the predictors for a skill score A or B, we performed a univariate analysis, considering as predictor factors: age (≥median), provenience (from Africa), marital status (single), level of education (S or U), level of knowledge (score A or B of the questionnaire). Double entry tables were constructed, χ2 and Odds Ratio (OR) with 95% confident interval (95% CI) were calculated, considering p < 0.05. To control the confounders, we also performed a multivariate analysis by a logistic regression model, considering as independent variables the previous described factors and p < 0.05. Data were analysed by using STATA 14.1 SE for Mac Os.

## Results

Characteristics of the study sample are reported in Table 1.

**Table 1:** Sample description

	N.	%
Age		
≥Median age (26 years)	42	52.5
<Median age (26 years)	38	47.5
Provenience		
Africa	54*	67.5
Asia	26**	32.5
Marital status		
M	53	66.3
S	27	33.7
Years of instruction		
E	15	18.7
M	28	35
S	30	37.5
U	7	8.8
Questionnaire		
A	40	50
B	30	37.5
C	6	7.5
D	4	5
Skill score		
A	22	27.5
B	33	41.3
C	18	22.5
D	7	8.7

* N. = 2 Ciad, N. = 6 Costa d’Avorio, N. = 3 Eritrea, N. = 11 Ghana, N. = 4 Mali, N. = 2 Mozambico, N. = 13, Nigeria, N. = 5 Senegal, n. = 8 Somalia

** N. = 7 Afghanistan, N. = 1 Giordania, N. = 10 Iraq, N. = 5 Siria, N.3 = Turchia

Migrants’ median age was 26 years-old (range 18–44) and the sample was composed of 80 persons: 54 (67.5%) from sub-Saharan Africa and 26 (32.5%) from Middle East. Among Africans, migrants prevalently came from Nigeria (N. = 13), then from Ghana (N. = 11), Somalia (N. = 8), Côte d’Ivoire (N. = 6), Senegal (N. = 5), Mali (N. = 4), Eritrea (N. = 3), Chad (N. = 2) and Mozambique (N. = 2). Middle East migrants landed from Iraq (N. = 10), Afghanistan (N. = 7), Syria (N. = 5), Turkey (N. = 3) and Jordan (N. = 1). Most of the migrants were married (N. = 53, 66.25%), with a lower middle level of education, up to 8 (N. = 28, 35%) or 5 years (N. = 15, 18.75%). However, 30 Borgo Mezzanone’s guests (37.5%) studied more than 8 years and 7 persons (8.75%) attended university courses.

Only 10 migrants scored C (N. = 6, 7.5%) or D (N. = 5%) in the questionnaire, while half of the sample (N. = 40, 50%) achieved class A. Remaining 30 migrants (37.5%) scored B. Finally, our skill score showed a large number of people in class A (N. = 22, 27.5%) or B (N. = 33, 41.25%), while class C (N. = 18, 22.5%) or D (N. = 7, 8.75%) were less represented.

The Spearman’s rho was 0.30, therefore answers to the questionnaire and skill score appeared correlated (p < 0.05).

In the univariate analysis, to have a skill score A or B resulted to be associated to a level of education S or U (OR = 7.9, 95% CI 2.2–35; p < 0.001), and it’s linked to a score A or B in the questionnaire (OR = 6.7, 95% CI 1.3–43.4; p < 0.05; power of estimation = 68.1%—Table 2).

**Table 2:** Univariate analysis

	Skill score A or B (N. = 55)		Skill score C or D (N. = 25)		χ^2^	p	OR	IC
	N.	%	N.	%				
Age								
<Median age (26 years)	30	54.55	8	32			ref.	
≥Median age (26 years)	25	45.45	17	68	3.5	>0.05	2.6	0.9–8
Provenience								
Asia	19	34.55	7	28			ref.	
Africa	36	65.45	18	72	0.34	>0.05	0.7	0.2–2.3
Marital status								
M	37	67.27	16	64			ref.	
S	18	32.73	9	36	0.08	>0.05	0.9	0.3–2.7
Years of instruction								
E or M	22	40	21	84			ref.	
S or U	33	60	4	16	13.39	*<0.001*	7.9	2.2–35
Questionnaire								
C or D	3	5.45	7	28			ref.	
A or B	52	94.55	18	72	7.99	*<0.05*	6.7	1.3–43.4

Italic values indicate significance of p value (p < 0.05)

In the multivariate model, to have a skill score A or B resulted to be associated to be older than 26 years (OR = 5.8, 95% CI 1.3–26.9; p < 0.05), with a level of education S or U (OR = 10.4, 95% CI 2.6–41.9; p = 0.001), and a score A or B in the questionnaire (OR = 11.4, 95% CI 1.6–80.4; p < 0.05—Table 3).

**Table 3:** Multivariate analysis

	OR	z	p	95% CI
Age (≥median age 26 years)	5.8	2.25	*0.025*	1.3–26.9
Provenience (Africa)	1.7	0.81	0.418	0.5–5.9
Marital status (single)	4.3	1.78	0.076	0.9–21.2
Years of instruction (Superior or University)	10.4	3.28	*0.001*	2.6–41.9
Questionnaire (A or B)	11.4	2.44	*0.015*	1.6–80.4

Italic values indicate significance of p value (p < 0.05)

## Discussion

Worldwide, a number of factors associated with migration place migrant populations at higher risk for HIV infection, including extreme poverty, substance and alcohol abuse, obstacles with different language and culture, migration patterns, immigration status, disparities in access to health care, segregation and social exclusion, and lack of education [9, 10].

Furthermore, migration has been associated with higher levels of depression, loneliness, and isolation as well. The sense of solitude and isolation experienced by migrants may be the driving force behind embracing unhealthy behaviors, such as practicing risky sexual practices [11, 12]. These populations may become a reservoir of latent infections because of poor health care in their native Countries or because of social and ethnic marginalization [13–17].

Efforts to prevent the transmission of HIV have largely focused on education and behavior change, but several studies reported scarce use of condom among migrants [18–21].

HIV/STI prevention interventions should focus on promoting consistent as well as correct use of condoms because their improper use is associated to failure rate, due to improper application or removal, or both, other than breakage or slippage and timing of application and removal (as well as the skills involved) [22].

Studying the general level of Condom Use Skills in our sample, composed of migrants recently landed in Italy, we find no significant differences in the level of Condom Use Skills between African and Middle Asian migrants.

We find that level of education and age are associated with better answers to the questionnaire and an high skill score. For these reasons, we suppose that education level can influence the quality of advice and knowledge imparted and have contributed to condom use behaviours and level of Condom Use Skills. Furthermore, other studies underline the role of better-educated friends or acquaintances’ communication in improving Condom Use Skills [24].

Single or married men of our sample don’t show relevant differences in the answers: this is an important remark because other studies suggest that low condom skill score may be related to single status or living as temporary single condition [25].

Although a low skill score may not predict absolute failure of HIV infection prevention, it would be advisable to proceed with caution in using this score as a means of predicting real-life condom use, about 30% of our sample results in class C or D, an alarming percentage. It would be important to have a comparative norm, but literature about vulnerable populations is scarce, while other categories, such as female sex worker (FSW), reached important goals in the prevention of STIs and HIV with a higher condom use [29–32]. On the other side, a longitudinal research in an urban canadian setting highlights the persistent challenges faced by migrant sex workers in terms of accessing and using condoms [26].

Our sample is only composed of asylum-seekers: although Italian residence permit requests are justified mainly by business employment (48%) or familiar reunification (41%), in 2014 the third most frequent cause is asylum-seeking and humanitarian aid (5%), overtaking studying reasons [1]. The observed skills of condom use among our participants may be different from other populations, such as foreigner sex workers, and this could be a limitation of our study.

Another critical issue is the presence of only men in our sample, but another study that performed additional analyses for males and females separately showed no significant differences in demonstrated condom skills [22]. However, it would be interesting to carry out a new study to make comparisons between male and female migrants.

As peer reviewers had suggested, different methods for classifying and analyzing data in this study could have be adopted. We have chosen to assign one point for each value in the questionnaires used to evaluate the attitudes, knowledge and skills to simplify statistical analysis. According to the assumption that all items are equally important, the dichotomist classification reduced the variability and gave the immediate overview on the critical targets to be included in specific and rapid interventions. Nevertheless, further evaluations using quantitative methods, alongside scrutiny of each item on the skill scales, will be able to supply more nuanced and detailed insight into this complex situation and to identify more tailored health promotion requirements.

After all, recent researches are also focusing on the timing of condom application (late application or early removal as being crucially important aspects), but we don’t include these features because it’s difficult to see how they could be with a wooden penile model [27, 28].

## Conclusions

The main outcome of our study is the correlation between answers to the questionnaire and skill score, both strongly influenced by level of education. However, we can’t ignore that more of the half of our sample have studied less than 9 years, with an high risk of misunderstanding campaigns for distribution of informative materials. The presence of cultural mediators could be not enough if migrants aren’t conscious of behavioral risks and don’t understand how to prevent STIs, such as HIV infection. The lack of sanitary operators that speak fluently different languages is surely a side of the problem but it’s important to improve assistance-related social support measures and to increase migrants’ literacy. For example, interventions targeted at reducing the spread of HIV should, in addition to promoting the consistent use of condoms, include instruction on the effective use of these items, without forget to consider the psychosocial and situational barriers associated with this method of protection.

Finally, our study underlines the importance of helping illiterate or poor schooled migrants, that represent a vulnerable and marginalized group necessitous of social support, that should focus on promoting guidance, advice, knowledge and information on the correct use of condoms.

## Additional file

**Additional file 1.** Questionnaire (English version).

## Abbreviations

STI: sexual transmitted infecion
CUS: Condom Use Skills
OR: odds ratio
CI: confident interval
