# Supplementary material for: Correlation between knowledge on transmission and prevention of HIV/STI and proficiency in condom use among male migrants from Africa and Middle East evaluated by a Condom Use Skills score using a wooden penile model
Source: BMC Res Notes. 2017 Jun 19;10:216. doi: 10.1186/s13104-017-2520-1 (PMC5477424; doi:10.1186/s13104-017-2520-1)
Supplement: Supplementary file 1 — Additional file 1. Questionnaire (English version). [file 13104_2017_2520_MOESM1_ESM.docx]

**Questionnaire (English version)**

1. Which of the following diseases can be transmitted through sexual intercourse?

a) Syphilis

b) AIDS

d) Malaria

e) A+B

f) All answers are correct

2. Which of these contraceptive methods prevent sexually transmitted diseases?

a) male condom

b) female condom

c) Intra Uterine Device

d) spermicidal cream

e) A+B

f) All answers are correct

3. Unprotected sex:

a) It can cause sexually transmitted diseases only in cases of vaginal penetration

b) It can cause sexually transmitted diseases only if the infected partner reaches orgasm

c) in case of oral intercourse there is no risk of infection

d) A+B

e) All answers are correct

f) None of the answers is correct

4. The chances of HIV infection due to unprotected sex are:

a) Higher for women

b) Higher for men

c) Equal for both sexes

d) Extremely low if sexual intercourse is not accompanied by the use of drugs

e) Facilitated by the simultaneous presence of other STDs

f) A+E

5. Globally, the majority of HIV infections are transmitted:

a) by heterosexual contacts

b) by homosexual contacts

c) by infected blood transfusions

d) by common use of infected syringes

e) from mother to child

6. A partner who does not have symptoms suggestive of sexually transmitted diseases:

a) He/she has a particularly strong immune system that protects him/herself from STDs

b) He/she could be a healthy carrier of an STD, so he/she can not in any way pass it on to the partner

c) He/she could be a healthy carrier of an STD, so he/she can pass it on to the partner

7. If an STD is diagnosed, the treatment prescribed by the doctor to eradicate the infection:

a) It is reserved exclusively for persons affected by MST

b) It must be extended to the partners with whom the individual had unprotected sex

c) It must be extended to the partners with whom the individual had unprotected sex, only if symptoms suggestive of the disease

d) None of the answers is correct

8. The use of condoms:

a) It provides absolute protection from STDs, if worn properly

b) It offers excellent protection from STDs, provided that a proper fit. However, some STDs can also be transmitted using a condom

c) Does not protect against STDs areas not covered, they may be at risk of STDs (eg genital warts)

d) A+C

e) B+C

9. Which of the following sexual practices is associated with LOWER risk of contracting a sexually transmitted disease?

a) oral sex

b) vaginal sex

c) anal sex

10. Which of the following sexual practices is associated with HIGHER risk of contracting a sexually transmitted disease?

a) oral sex

b) vaginal sex

c) anal sex

11. Coitus interruptus:

a) Effectively protects against sexually transmitted diseases

b) Effectively protects against unwanted pregnancies

c) both the answers are correct

d) None of the answers is correct

12. The use of birth control pill:

a) It provides good protection from STDs

b) It is more effective than condoms in protecting against STDs

c) both the answers are correct

d) None of the answers is correct

13. Practicing sex in a pool or in a bathtub:

a) If the water is hot enough (> 40 ° C) protects against sexually transmitted diseases

b) It protects against STDs with bactericidal action of chlorine in drinking water and in one of the pools

c) If equipped with a whirlpool, the air introduced into the water kills the bacteria responsible for STDs

d) A+B

e) None of the answers is correct

14. If for the past years there have been multiple sex with several partners without experiencing any symptoms of STDs:

a) It means that the immune system is strong and able to protect against STDs

b) the possibility of suffering from some STDs or their complication in asymptomatic stage is not excluded

c) Although they can suffer from an asymptomatic STDs, you can not pass it on to other people.

15. After being healed from a sexually transmitted disease:

a) The immune system protects for many years from that specific STD

b) The immune system protects for many years by all STDs

c) You can not re-contracting the same disease for life

d) All answers are correct

e) None of the answers is correct

16. HIV is:

a) a virus

b) a fungus

c) a parasite

17. Where can you buy condoms?

a) at the supermarket

b) in pharmacies

c) in vending machines

d) All answers are correct

18. If a condom breaks during sex:

a) there is risk of contracting sexually transmitted diseases

b) there is a risk of unwanted pregnancies

c) there is no risk if sexual intercourse was short

d) A+B

19. What is the right time to wear a condom?

a) before each sexual intercourse, regardless by erection

b) during each sexual intercourse, only at the time of erection

c) it does not matter when, just use it

20. In case of paid sex:

a) if sex worker agrees, you can not use condom without risks

b) condom is useless because sex workers are strictly controlled

c) condom is always useful
